# Electron transport through supercrystals of atomically precise gold nanoclusters: a thermal bi-stability effect†

**DOI:** 10.1039/d3sc02753h

**Published:** 2023-11-09

**Authors:** Tatsuya Higaki, Jake C. Russell, Daniel W. Paley, Xavier Roy, Rongchao Jin

**Affiliations:** a Department of Chemistry, Carnegie Mellon University Pittsburgh Pennsylvania 15213 USA rongchao@andrew.cmu.edu; b Department of Chemistry, Columbia University New York New York 10027 USA xr2114@columbia.edu; c Columbia Nano Initiative, Columbia University New York New York 10027 USA

## Abstract

Nanoparticles (NPs) may behave like atoms or molecules in the self-assembly into artificial solids with stimuli-responsive properties. However, the functionality engineering of nanoparticle-assembled solids is still far behind the aesthetic approaches for molecules, with a major problem arising from the lack of atomic-precision in the NPs, which leads to incoherence in superlattices. Here we exploit coherent superlattices (or supercrystals) that are assembled from atomically precise Au_103_S_2_(SR)_41_ NPs (core dia. = 1.6 nm, SR = thiolate) for controlling the charge transport properties with atomic-level structural insights. The resolved interparticle ligand packing in Au_103_S_2_(SR)_41_-assembled solids reveals the mechanism behind the thermally-induced sharp transition in charge transport through the macroscopic crystal. Specifically, the response to temperature induces the conformational change to the R groups of surface ligands, as revealed by variable temperature X-ray crystallography with atomic resolution. Overall, this approach leads to an atomic-level correlation between the interparticle structure and a bi-stability functionality of self-assembled supercrystals, and the strategy may enable control over such materials with other novel functionalities.

## Abstract 2

Supercrystals assembled from atomically precise gold nanoclusters exhibit a thermally responsive transition in charge transport.

## Graphical abstract

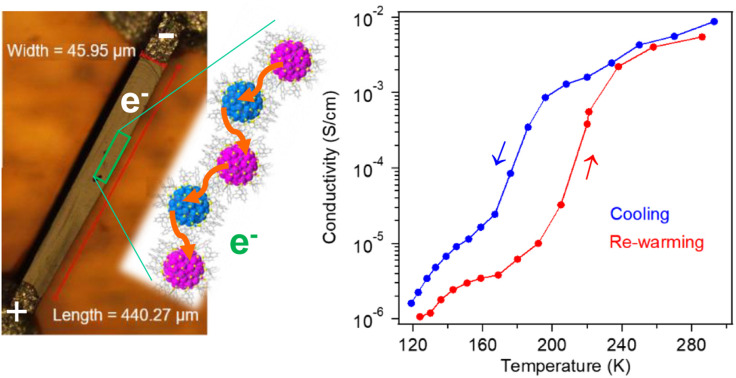

## Introduction

Stimuli-responsive materials of molecules or organometallics can show dramatically improved functionalities owing to the tailoring strategy by molecular chemistry.^1^ In contrast, the functionality engineering of self-assembled colloidal nanoparticles (NPs)^2^ still lags behind the aesthetic approaches for molecules, although research on the NP systems has made tremendous progress in controlling the packing structures^2–4^ as well as surface functionalization for versatile nanomaterials.^5–7^ The major problem hindering the realization of molecular-precision assembly of NPs arises from the lack of atomic-precision of those NPs in both the core and the surface shell of ligands. While much work on charge transport in nanoparticle assemblies has been reported, the inherent size dispersity of conventional NPs (*e.g.* standard deviation of ∼5%) and their elusive surface structure preclude the attainment of atomically coherent superlattices (*i.e.* supercrystals), because the coherence from the crystalline cores of NPs to the nanometer periodicity of superlattices is destroyed by the rotation of NPs and random packing of interparticle ligands in the NP assemblies.^8^ Therefore, no atomic-level insight could be achieved.^8,9^

It has long been a major dream to obtain atomically precise NPs and assemble them into atomically coherent superlattices for achieving versatile functionalities.^5,6,10^ Recent advances in nanochemistry^11–17^ have offered access to gold NPs with atomically precise cores and definitive numbers of surface ligands (*e.g.*, thiolate –SR or phosphine PPh_3_),^10,16,17^ which are represented by exact formulae (*e.g.*, Au_*n*_(SR)_*m*_ for thiolate-protected NPs), akin to molecules.^18–21^ Such ultrasmall NPs (1–3 nm core diameter) are often called nanoclusters (NCs). More importantly, crystallization of such NCs can lead to long-range coherence from the angstrom level to the macroscopic length scale (*e.g.*, millimeter) in the assemblies,^22–24^ which offers new opportunities in transport measurements, including photoconductivity^25^ and electrical conductivity.^26–29^ The structural order is very important in charge transport.^27^ Yuan *et al.*^28^ discovered an anisotropic effect (in plane *vs.* out of plane) in supercrystals assembled from nanoclusters. In recent work, Zhu *et al.*^29^ demonstrated an electronic spin-polarized transport (*i.e.* a spin valve effect) in assembled nanoclusters by observing a magnetoresistance of 1.6% even at room temperature, which was attributed to the spin–orbit coupling (SOC) effect of NCs.

Here we report a thermally-induced sharp transition (*i.e.* a bi-stability effect) in electron transport through supercrystals self-assembled from atomically-precise gold NCs, including Au_103_S_2_(SR)_41_, Au_133_(SR)_52_, and Au_144_(SR)_60_. As for Au_103_S_2_(SR)_41_, the interparticle ligand packing structure is rationalized to be responsible for the thermally responsive transition of charge transport through the atomically coherent crystals. X-ray crystallography with atomic resolution (0.8–1.1 Å) reveals that the response to external temperature induces conformational changes to the naphthalene groups of surface ligands. The obtained structural insights reveal a correlation between the surface structure and functionality of self-assembled supercrystals with atomically-precise gold NPs. The strategy described here will help material scientists to improve the tailoring method for atomic-level control over nanostructures and solid materials for novel functionalities.

## Results and discussion

The atomically precise Au_103_S_2_(SR)_41_, Au_133_(SR)_52_, and Au_144_(SR)_60_, where the R groups are 2-naphthalene, 4-*tert*-butyl benzene, and benzyl group, respectively, were synthesized by “size-focusing” and/or “ligand-exchange” methods,^30–32^ followed by supercrystal growth. The as-prepared crystals were then used for current–voltage (*I*–*V*) measurements at variable temperatures (from 300 K down to 120 K) to study thermal-responsive conductivity of the NC self-assembled crystal. The stimuli-responsive conductivity is further correlated with the structural changes by performing X-ray crystallography at different temperatures.

### Charge transport through the Au_103_ supercrystal

**Fig. 1:**
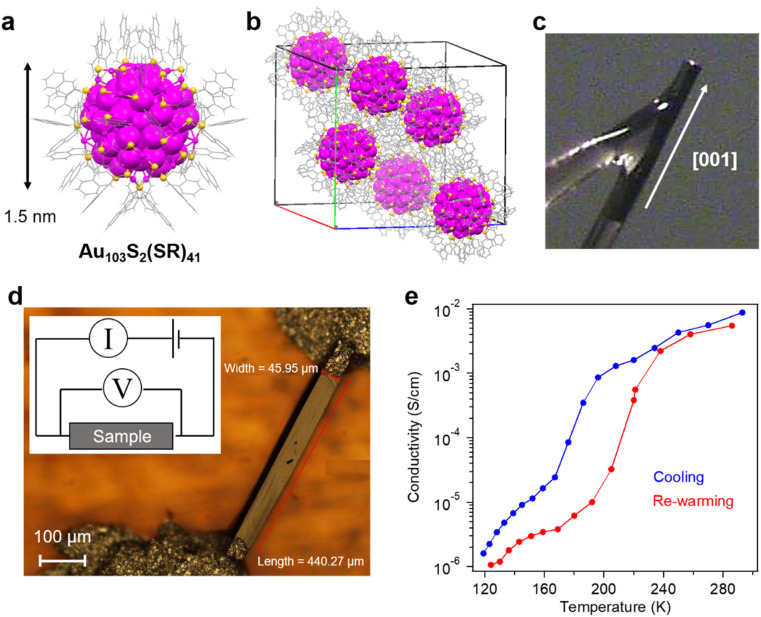
Self-assembled crystal of atomically-precise Au_103_ NCs for charge transport measurement. (a) Crystal structure of Au_103_S_2_(SR)_41_. (b) Unit cell of a single crystal of Au_103_. (c) Face indexing of a Au_103_ crystal to determine the long axis. (d) Charge transport study on a Au_103_ assembled crystal along the [001] direction. (e) Conductivity of a Au_103_ assembled crystal at variable temperatures.

We first discuss the charge transport properties of the Au_103_S_2_(SR)_41_-assembled crystals (Au_103_ for short). The synthesis of atomically-precise Au_103_ was performed by a ligand-exchange reaction from Au_99_(SPh)_42_ with 2-naphthalenethiol.^30^ A vapor diffusion of methanol into 1,2,4-trichlorobenzene solution of Au_103_ yielded needle-shaped crystals. Each Au_103_ NC shows an atomically-precise structure made up of a Au_79_ core protected by oligomeric –S–(Au^I^–S)_*n*_– motifs which can be called “staples” (Fig. 1a), and the crystal exhibits a rod-like morphology (Fig. 1b and c). The crystal is in a monoclinic crystal system with *C*2/*c* space group. Crystallographic face indexing determined the longitudinal axis of the crystals as [001], thus, the charge transport measurements were performed along the [001] axis of single crystals (Fig. 1b and c). Both ends of the crystal were covered by silver paste in order to form good contacts (Fig. 1d). At room temperature, the conductivity (*σ*) of a typical Au_103_ crystal was ∼10^−2^ S cm^−1^. Interestingly, it drastically decreased to ∼10^−6^ S cm^−1^ at 120 K with a sharp drop (∼two orders of magnitude) over 196–167 K (Fig. 1e).

Upon re-warming, the conductivity gradually increases with increasing temperature to 192 K, followed by a sharp increase of conductivity over 192 to 238 K. The hysteresis loop shows a two-orders-of-magnitude difference at ∼180 K (mid-point) for cooling and at ∼210 K for re-warming (Fig. 1e). Such a hysteresis is reversible, indicating no thermally caused damage to particle arrangements in the crystal or other irreversible processes.

The conductance in the Au_103_-assembled crystal increased with temperature, indicating that thermal activation (*k*_B_*T*) is required to overcome the energy barriers between NCs.^33,34^ This is typical of the charge hopping mechanism,^35,36^ and the activation energy is expressed by the equation:

1 *σ*(*d*, *T*) = *σ*_0_e^−*βd*^e^−*E*_A_/*k*_B_*T*^

where, *d* is the interparticle distance (Å), *T* is the temperature (K), *β* is the electron-tunneling coefficient, and *E*_A_ is the activation energy (eV). A system with weak electronic coupling is known for this type of conducting behavior (such as the highly disordered organics or NPs' film).^37–40^ Therefore, our observation indicates that the charge carriers are localized in each NP, resulting in smaller interparticle electronic coupling (*β* ≪ *k*_B_*T*). The plot from eqn (1) shows that the activation barriers in the Au_103_ crystal varied in different temperature ranges (see Fig. S1†), with the activation energies determined to be I: 0.12 eV (293–196 K), II: 0.35 eV (196–167 K), and III: 0.093 eV (167–119 K). These values are comparable to *E*_A_ values in weakly coupled organic crystals.^41^

### Sizes and energy-gaps of nanoclusters

To investigate the potential NC size and bandgap (*E*_g_) effects, we compared the charge transport properties of Au_103_ crystals with other crystals assembled from Au_133_(SR′)_52_ or Au_144_(SR′′)_60_ (Fig. S2 and S3†). All these are thiolate-protected (with >100 Au atoms) and show clearly resolved carbon tail structures by X-ray crystallography (Fig. S2 and S3†). Under the same measurement conditions, the crystals exhibited a trend of decreasing conductivity with cooling, and the room temperature conductivity is similar (∼10^−2^ S cm^−1^) among the crystals of 3 sizes of NCs, indicating negligible effects of energy-gaps of each type of NCs (note: the *E*_g_ varies from 0.5 eV of Au_103_ to 0.1 eV of Au_144_); thus, the core sizes are less critical, rather the ligands should play a more important role. Among the three systems, the naphthalenethiolate-protected Au_103_ crystals showed the most distinctive thermal responsivity (width of the hysteresis: ∼40 K, Fig. 1e), while the Au_144_(SR′′)_60_ crystal (where R′′ = benzyl group) showed a similar two-orders of magnitude transition but a much less prominent hysteresis due to less extensive C–H⋯π interaction of the smaller benzene ring (Fig. S3b†) than the naphthalene groups on Au_103_ (Fig. S4†), and the case of Au_133_(SR′)_52_ (R′ = 4-*tert*-butyl benzene) exhibited neither transition nor hysteresis due to the almost destroyed C–H⋯π interaction by the *tert*-butyl group on the benzene ring (Fig. S2b†). Overall, the observed trend implies the important role of the ligand type and the interparticle ligand packing in the crystal.

### Variable temperature single crystal XRD analysis

**Fig. 2:**
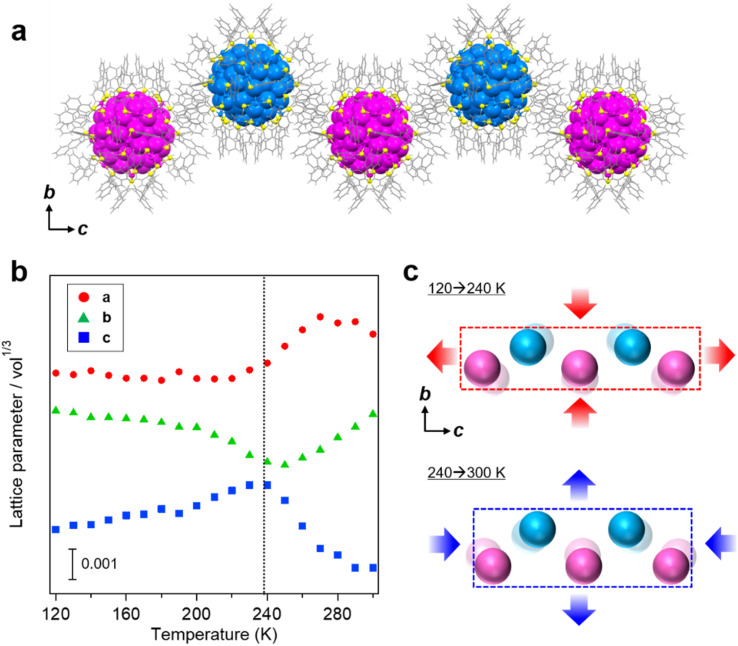
Variable temperature SCXRD analysis of a Au_103_-assembled crystal. (a) Packing of Au_103_ NCs along [001] direction (the *c* axis) in the crystal. Blue and magenta illustrate Au atoms in different Au_103_ enantiomers. (b) Unit cell parameters at 120–300 K normalized by cell volume. (c) Schematic illustration of rearrangement of Au_103_ NCs while re-warming.

In order to obtain further structural insight into the thermally-induced sharp transition and hysteresis in the Au_103_ crystal, variable temperature single crystal XRD (SCXRD) was performed (Fig. 2). We find that Au_103_ NCs are linearly assembled along the [001] direction of the crystal, which is the same direction as the charge transport (Fig. 2a). The metal core structures do not show any change during the cooling/rewarming processes. Fig. 2b summarizes temperature-dependent unit cell lengths normalized by cell volume. Upon re-warming from 120 K, the crystal experiences an elongation along the [001] direction, which continues with the temperature rise to 240 K. Above 240 K, the unit cell starts to shrink along the [001] direction until reaching room temperature. The unit cell length along the *b*-axis shows opposite changes: a gradual decrease between 120 K and 240 K but an increase between 240 and 300 K. The schematic illustration of NC arrangement is shown in Fig. 2c. It is interesting to observe that the unit cell parameters for *b*- and *c*-axis show volcano-like behavior with the transition temperature at ∼240 K upon re-warming (Fig. 2b). This temperature is indeed comparable to the temperature of sharp conductivity changes during the re-warming process. Thus, the correlation between the observed temperature-induced changes for unit cell parameters and conductivity suggests that the sharp conductivity transition should be induced by the interparticle conformational change in response to external heat. Previous work on charge transport in NP assemblies^42,43^ could not observe any sharp transition possibly because of the polydispersity of NPs and lack of atomic-level coherence. In our system, we observed a two-orders-of-magnitude variation in conductivity over the sharp transition and distinct hysteresis behavior in the atomically coherent assembly of precise NPs. The underlying mechanism is revealed by resolving interparticle ligands' conformation.

The conformation-induced transition/hysteresis mechanism is also supported by the much less distinct behavior in the cases of Au_133_ or Au_144_ crystals that do not show a linearly-assembled wire-like structure with interlocked aromatic carbon tails of naphthalene groups; note that the potential interlocking of *tert*-butylbenzene groups in the Au_133_ sample is destroyed by *tert*-butyl, whereas the benzyl groups in the Au_144_ sample can partially interlock but much less than the naphthalene case. Therefore, the distinct transition/hysteresis in Au_103_ crystals should originate from the linearly assembled Au_103_ NCs along the [001] direction, which is the direction of the measured electrical conductance, and the conformational changes to interparticle naphthalene groups along the [001] induces the sharp transition due to the phase transition between the less conductive conformation (yet more stable) at low-T and the more conductive conformation at high-T. While molecular packing is involved in the charge transport properties of organic crystals,^44–46^ the systems of thiolate-protected NCs are much more complex due to the inorganic/organic hybrid and spherical topology (as opposed to planar organic molecules, which involves relatively simple π–π stacking interactions). In randomly assembled films of atomically precise NCs^25,26^ or coherent crystals without interlocked aromatic groups,^27,28^ no coherent effect of ligands was observed. In order to resolve the changes with atomic resolution, we further performed full-structural analysis by X-ray crystallography at 210 and 300 K, respectively, and the obtained insights are discussed below.

**Fig. 3:**
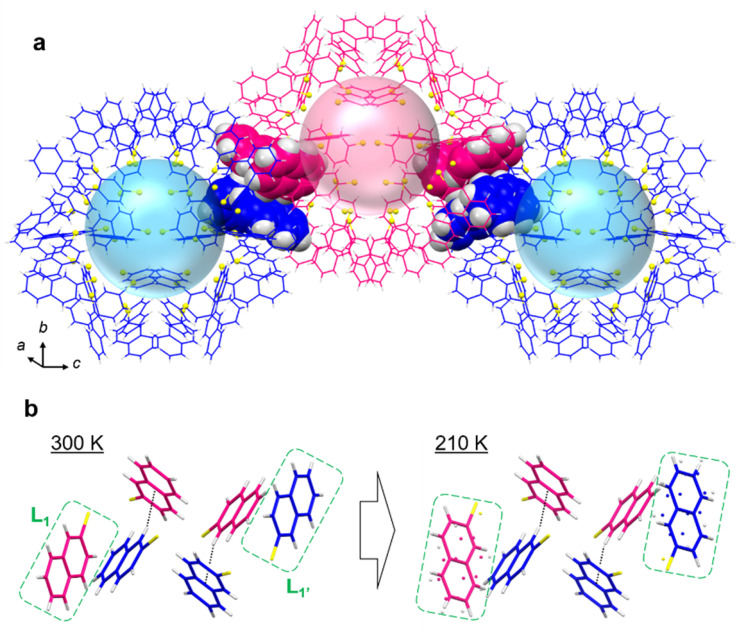
Carbon tail structures between self-assembled Au_103_ NCs. (a) Packing of Au_103_ along the [001] direction with highlights in interlocked naphthalene carbon tails between adjacent NCs. (b) Temperature-dependent conformation changes in interparticle C–H⋯π interactions for interlocked naphthalene carbon tails.

First of all, ample C–H⋯π interactions are found in the crystal of Au_103_ NCs. The intra-particle C–H⋯π distance is 2.58 ± 0.08 Å, which is considerably shorter than 2.73 ± 0.13 Å for typical C–H⋯π distance in organic crystals.^47^ In addition, the Au_103_ NCs are linearly assembled *via* C–H⋯π interaction between the surface-protecting 2-naphthalenethiolate ligands (Fig. 3a). Of note, the interparticle distance (core center-to-center) is 23.74 Å along the *c*-axis (Fig. S4†), while for other directions the nearest distances (27.41 Å and 27.48 Å) are significantly longer. Thus, the charge hopping should largely proceed along the *c*-axis or [001] direction. Charge carriers are considered to travel through the interlocked naphthalene groups as highlighted in Fig. 3a during the charge transport along the [001] axis. At 300 K, the interparticle ligands' C–H⋯π interactions show a distance of 2.920 Å, which increases to 2.941 Å at 210 K, hence, the conductivity drops at low temperatures. Further insights into the conformational changes are obtained for adjacent ligands (noted as L_1_ and L_1′_) near interlocked ligands (Fig. 3b). Interestingly, at 210 K, conformational disorders are observed for these neighboring ligands, which are not observed at 300 K. This observation suggests that the decrease in conductivity is induced by the conformational changes of interparticle ligands' interactions, rather than the interparticle distance (invariant at 210 K and 300 K). Although the observed structure difference does not seem large, more dramatic changes are expected to be observed at much lower temperatures considering the significantly lower conductivity (see above Fig. 1e).

We note that the non-linear assembly in the cases of Au_133_ and Au_144_ crystals and their lack of strong inter-locking pattern of ligands lead to significantly less distinct transition and hysteresis in the crystals of Au_133_ and Au_144_ compared to the naphthalenethiolate-protected Au_103_ NCs.

### Thermal analysis on the transition

**Fig. 4:**
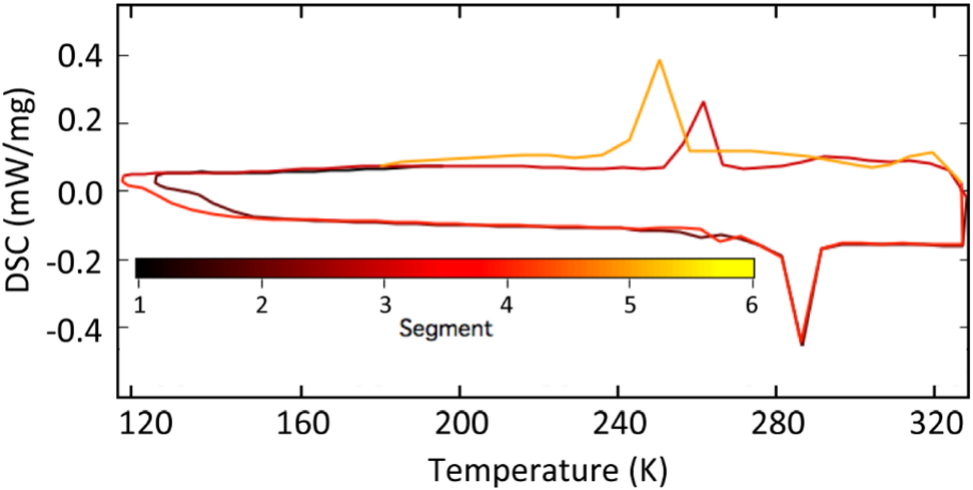
DSC analysis of the phase transition in the supercrystal assembled from Au_103_ NCs.

We further carried out differential scanning calorimetry (DSC) analysis between −150 and 50 °C (123−323 K, Fig. 4). Two reversible transformations were observed in the crystal assembled from Au_103_ NCs, and the transition observed in DSC occurs near the temperature of the transition in the conductivity measurements, indicating that the electron transport change was induced by the phase transition, consistent with the X-ray crystallographic analysis.

## Conclusions

In summary, this work presents the discovery of thermally induced sharp transition and hysteresis of conductivity in crystals assembled from atomically precise Au_103_ NCs and reveals atomic-level insight into the structural correlation with charge transport properties. The crystals exhibit charge hopping transport with decrease in conductivity upon cooling, due to the weak interparticle electronic coupling, and abrupt changes in activation energy are revealed by Arrhenius-like plots. Further crystallographic analysis rules out the interparticle distance change and unravels the conformational changes to the interparticle ligands, the latter explains the temperature dependent conductivity as well as the thermal hysteresis. This work elucidates the critical role of interparticle ligands' conformation on the charge transport of assembled NPs with atomic-level coherence. The bi-stability behavior of coherent crystal materials of nanoclusters *via* ligand tailoring may hold potential in future exploration for memory, sensing, and actuation applications.

## Data availability

All the data are included in the ESI; Crystallographic structure files (cif deposition numbers: 2 153 700 for Au_103_–300 K, 2 153 701 for Au_103_–210 K) can be retrieved at CCDC, https://www.ccdc.cam.ac.uk.

## Author contributions

T. H. and J. C. R. contributed equally to this work. R. J. and X. R. conceived the project. T. H. performed all the synthesis and single crystal growth. J. C. R. conducted the variable temperature charge transport measurements. D. W. P. collected and analyzed X-ray diffraction data at different temperatures. T. H., J. C. R., X. R. and R. J. wrote the manuscript with the contributions from all the other authors.

## Conflicts of interest

There are no conflicts to declare.

## Supplementary Material

SC-014-D3SC02753H-s001
