# Supplementary material for: Electron transport through supercrystals of atomically precise gold nanoclusters: a thermal bi-stability effect
Source: Chem Sci. 2023 Nov 9;14(45):13191–7. doi: 10.1039/d3sc02753h (PMC10664525; doi:10.1039/d3sc02753h)
Supplement: SC-014-D3SC02753H-s001 [file SC-014-D3SC02753H-s001.pdf]

Electronic Supplementary Material (ESI) for Chemical Science.
This journal is © The Royal Society of Chemistry 2023

Supporting Information:

Electron Transport through Supercrystals of Atomically Precise Gold Nanoclusters: A Thermal Bi-stability Effect

Tatsuya Higaki,[a†] Jake C. Russell,[b†] Daniel W. Paley,[c] Xavier Roy,[b]* and Rongchao Jin[a]*

[a] Department of Chemistry, Carnegie Mellon University, Pittsburgh, Pennsylvania 15213;
[b] Department of Chemistry, Columbia University, New York, New York 10027;
[c] Columbia Nano Initiative, Columbia University, New York, New York 10027

Synthesis and crystallization. The synthesis and crystallization of Au nanoclusters (Au_{103}, Au_{133}, and Au_{144}) were performed according to the previously reported procedures as discussed in the main text.

Electrical conductivity measurement. Single crystals were selected and adhered to a thin copper plate. Contacts were made by painting to opposite sides of the needle-shaped crystal with conductive silver paint (Ted Pella 16032). Electrical transport properties were measured by applying a voltage sweep to the sample with a Keithley 2400 digital multimeter, amplifying the current with a Stanford Research Systems Model SR570 pre-amplifier, and recording the current with a Keysight 34401A digital multimeter. The temperature was controlled using a Lake Shore 331 digital temperature controller.

Two-probe electrical device was fabricated by adhering single crystals of the Au clusters to a thin copper plate using double-sided tape. Contacts were made with conductive silver paint (Ted Pella 16032). The device was transferred to a vacuum probe station. A voltage sweep from –1 to 1 V at a rate of 100 mV/s was applied and the current was measured to obtain current-voltage (I-V) curves. The temperature was varied between 290 and 120 K using a built-in heater, liquid N_2 and a temperature controller. The I-V curves were measured at fixed temperature after allowing at least 5 min for the temperature to equilibrate. A linear fit was applied to the I-V curves to extract the conductance values. Conductivity and resistivity were calculated by measuring the dimensions of each crystal with an optical microscope.

Single crystal X-ray diffraction (SCXRD) analysis. SCXRD data were collected on an Agilent SuperNova diffractometer using mirror-monochromated Cu Kα radiation. A needle-shaped black crystal with dimensions 0.28 × 0.07× 0.05 mm^3 was mounted in quick-drying clear nail varnish on a MiTeGen microloop to prevent solvent evaporation. The crystal was then held at the desired temperature with an Oxford Cryojet 700 with N_2 flow. Diffraction data were collected to 1.1 Å resolution at 300 K and 0.8 Å resolution at 210 K. Unit cell parameters were derived from all measured reflections. At 300 K, the monoclinic unit cell had dimensions a = 36.7530(8) Å, b = 41.254(14) Å, c = 44.0875(8) Å, β = 103.982(2) °. At 210 K, the monoclinic unit cell had dimensions a = 36.2023(7) Å, b = 41.1857(8) Å, c = 44.1204(9) Å, β = 104.399(2) °. In both cases, the centrosymmetric space group C2/c was confirmed by using starting coordinates from the previously determined structure. Data collection, integration, scaling (ABSPACK) and face-indexed absorption correction (numeric analytical methods[S1]) were performed in CrysAlisPro.[S2] It was possible to solve the structures by intrinsic phasing in ShelXT,[S3] but the best results were obtained by starting from the previously determined structure.[S4] Subsequent refinement was performed by full-matrix least-squares on F2 in ShelXL.[S5] Olex2[S6] was used for viewing and to prepare CIF files. PLATON[S7] was used extensively to model disordered solvent using SQUEEZE.[S8] ORTEP graphics were prepared in CrystalMaker.[S9] SC10H7 groups were modeled as rigid bodies with geometry taken from a high-quality literature structure (CSD ref-code BINTAT).[S10]

Details of crystallographic data and parameters for data collection and refinement are in Table S1 and S2. Thermal ellipsoids are rendered at the 50% probability level for anisotropically refined atoms; isotropically refined atoms are represented as circles. The thermal ellipsoid plots are necessarily simplified by omitting additional independent and symmetry-related positions of disordered atoms.

For 300 K dataset:

THETM01_ALERT_3_A: The value of sine(theta_max)/wavelength is less than 0.550 Calculated sin(theta_max)/wavelength = 0.4545

Response**:** The intensities outside 1.1 A resolution were essentially unmeasurable due to the extensive disordered solvent.

PLAT201_ALERT_2_A: Isotropic non-H Atoms in Main Residue(s) .... 200 Report C2_1 C3_1 C4_1 C5_1 C6_1 C7_1 etc.

Response: The data quality and extensive disorder did not permit anisotropic refinement of C atoms.

PLAT721_ALERT_1_A Bond Calc 1.43(17), Rep 1.39960 Dev... 0.03 Ang. C6B_12 -C7B_12 1.555 1.555 .... # 526 Check

PLAT721_ALERT_1_A Bond Calc 1.45(12), Rep 1.40840 Dev... 0.04 Ang. C9B_21 -C10B_21 1.555 1.555 .... # 740 Check

Response: This is a rounding error. The fractional coordinates of C atoms in the rigid SC10H7 groups are reported with a limited number of decimal places, and for poorly located groups (esp. with low occupancy) the rounding error is enough for the calculated (from atom coordinates) and reported (correctly, with full precision) distances to significantly mismatch.

PLAT973_ALERT_2_A Check Calcd Positive Resid. Density on Au4 3.53 eA-3
PLAT973_ALERT_2_A Check Calcd Positive Resid. Density on Au9 3.39 eA-3
PLAT973_ALERT_2_A Check Calcd Positive Resid. Density on Au12 3.39 eA-3
PLAT973_ALERT_2_A Check Calcd Positive Resid. Density on Au2 3.38 eA-3
PLAT973_ALERT_2_A Check Calcd Positive Resid. Density on Au18 3.21 eA-3
PLAT973_ALERT_2_A Check Calcd Positive Resid. Density on Au1 3.20 eA-3
PLAT973_ALERT_2_A Check Calcd Positive Resid. Density on Au7 3.17 eA-3
PLAT973_ALERT_2_A Check Calcd Positive Resid. Density on Au8 3.17 eA-3
PLAT973_ALERT_2_A Check Calcd Positive Resid. Density on Au11 3.03 eA-3
PLAT973_ALERT_2_A Check Calcd Positive Resid. Density on Au13 2.76 eA-3
PLAT973_ALERT_2_A Check Calcd Positive Resid. Density on Au5 2.67 eA-3
PLAT973_ALERT_2_A Check Calcd Positive Resid. Density on Au20 2.63 eA-3
PLAT973_ALERT_2_A Check Calcd Positive Resid. Density on Au32 2.61 eA-3

Author Response: The residual density is symmetrically distributed around all Au atoms and thus is probably caused by absorption.

For 210 K dataset:

PLAT029_ALERT_3_A _diffrn_measured_fraction_theta_full value Low . 0.915 Why?
Response: The crystal was coated in lacquer to slow down solvent loss and this caused difficulty in centering. Some runs were rejected for inconsistent scaling and the final integration is a balance between completeness and data quality.

PLAT201_ALERT_2_A Isotropic non-H Atoms in Main Residue(s) .... 200 Report C2_1 C3_1 C4_1 C5_1 C6_1 C7_1 etc.
Response: The data quality and extensive disorder did not permit anisotropic refinement of C atoms.

PLAT721_ALERT_1_A Bond Calc 1.39(18), Rep 1.42250 Dev... 0.03 Ang. C3_2 -C4_2 1.555 1.555 .... # 294 Check
PLAT721_ALERT_1_A Bond Calc 1.4(3), Rep 1.36060 Dev... 0.04 Ang. C5_2 -C6_2 1.555 1.555 .... # 298 Check
PLAT721_ALERT_1_A Bond Calc 1.4(3), Rep 1.35900 Dev... 0.04 Ang. C7_2 -C8_2 1.555 1.555 .... # 302 Check
PLAT721_ALERT_1_A Bond Calc 1.4(2), Rep 1.36390 Dev... 0.04 Ang. C10_2 -C11_2 1.555 1.555 .... # 307 Check
PLAT721_ALERT_1_A Bond Calc 1.82(13), Rep 1.78700 Dev... 0.03 Ang. S1B_21 -C2B_21 1.555 1.555 .... # 746 Check
PLAT721_ALERT_1_A Bond Calc 1.4(3), Rep 1.36050 Dev... 0.04 Ang. C5B_21 -C6B_21 1.555 1.555 .... # 754 Check
PLAT721_ALERT_1_A Bond Calc 1.4(4), Rep 1.35900 Dev... 0.04 Ang. C7B_21 -C8B_21 1.555 1.555 .... # 758 Check
PLAT721_ALERT_1_A Bond Calc 1.4(3), Rep 1.36400 Dev... 0.04 Ang. C10B_21 -C11B_21 1.555 1.555 .... # 763 Check
Response: This is a rounding error. The fractional coordinates of C atoms in the rigid SC10H7 groups are reported with a limited number of decimal places, and for poorly located groups (esp. with low occupancy) the rounding error is enough for the calculated (from atom coordinates) and reported (correctly, with full precision) distances to significantly mismatch.

Supplementary Figures:

Figure S1. Arrhenius-like plot and activation energies analysis for a Au_{103} crystal.

Figure S2. Self-assembled atomically-precise Au_{133} NPs for charge transport measurement. **a**, Crystal structure of $Au_{133}(SR')_{52}$ NP. **b**, Unit cell of a single crystal of Au_{133}. **c**, Charge transport study on a Au_{133} crystal. **d**, Conductivity of a Au_{133} crystal at variable temperature.

Figure S3. Self-assembled atomically-precise Au_{144} NPs for charge transport measurement. **a**, Crystal structure of $Au_{144}(SR')_{60}$ NP. **b**, Unit cell of a single crystal of Au_{144}. **c**, Charge transport study on a Au_{144} crystal. **d**, Conductivity of a Au_{144} crystal at variable temperature.

Figure S4. Interparticle distance in a Au_{103} crystal. a, Crystal packing from c-axis view. **b**, Model structure of **a** with interparticle distance. Color codes: gray = carbon; magenta and blue are used for Au and S in different enantiomers.

Figure S5. Variable temperature unit cell analyses by X-ray crystallography for a Au_{103} crystal.

Supporting Figures:

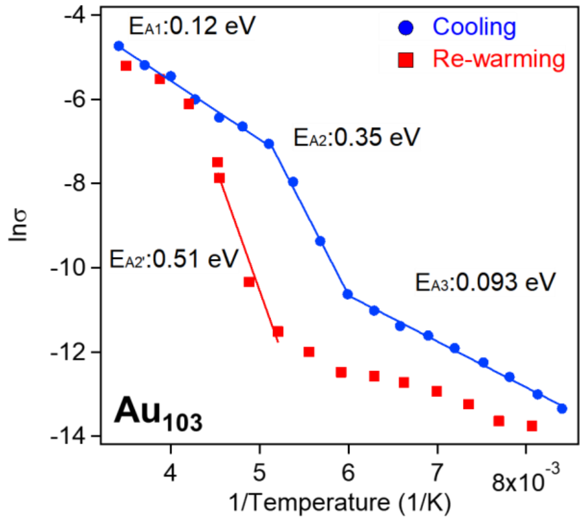

Figure S1. Arrhenius-like plot and activation energies analysis for a Au_{103} crystal.

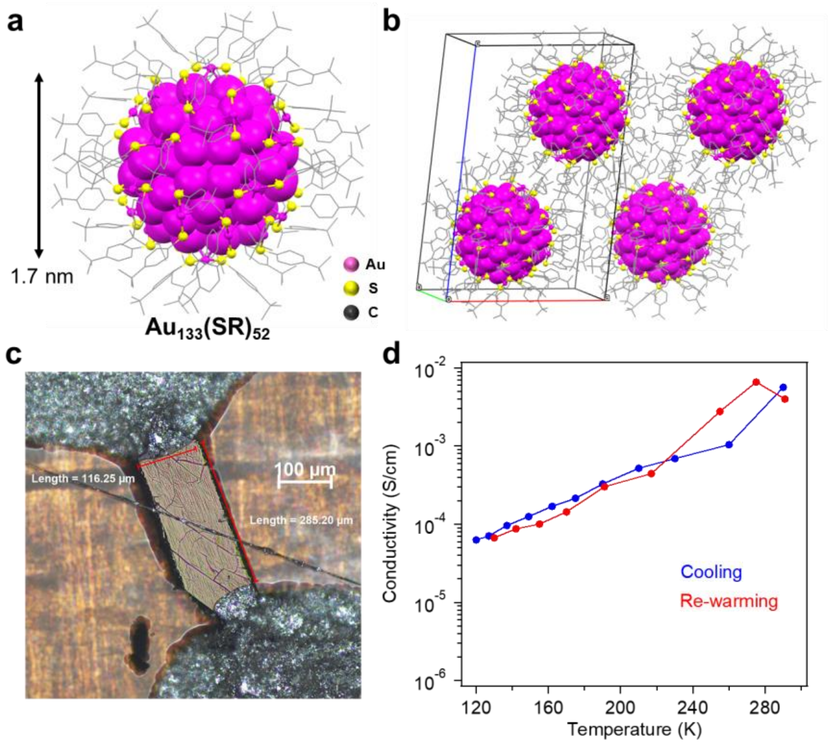

Figure S2. Self-assembled atomically-precise Au_{133} NPs for charge transport measurement. **a**, Crystal structure of $Au_{133}(SR')_{52}$ NP. **b**, Unit cell of a single crystal of Au_{133}. **c**, Charge transport study on a Au_{133} crystal. **d**, Conductivity of a Au_{133} crystal at variable temperature.

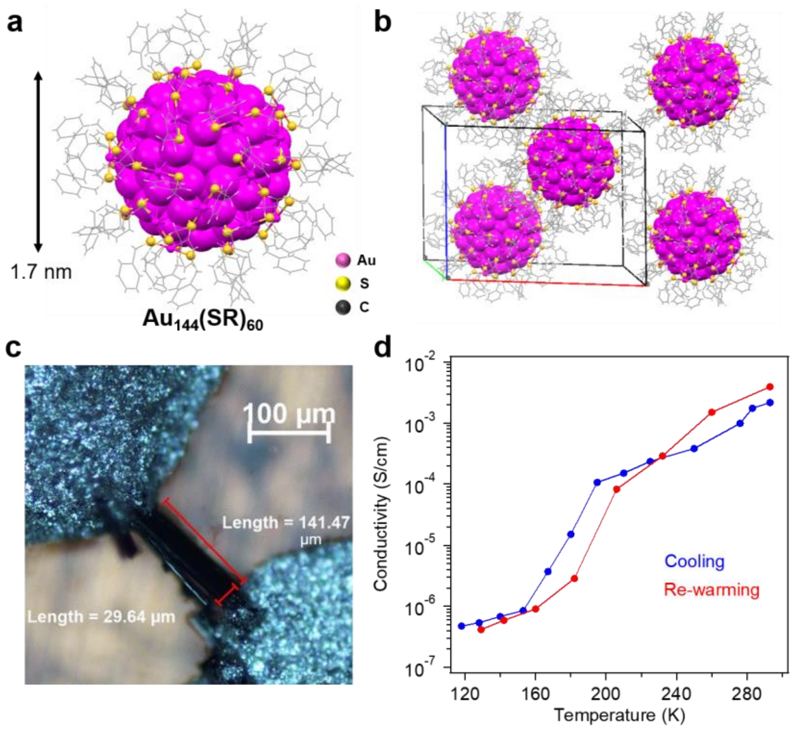

Figure S3. Self-assembled atomically-precise Au_{144} NPs for charge transport measurement. **a**, Crystal structure of $Au_{144}(SR')_{60}$ NP. **b**, Unit cell of a single crystal of Au_{144}. **c**, Charge transport study on a Au_{144} crystal. **d**, Conductivity of a Au_{144} crystal at variable temperature.

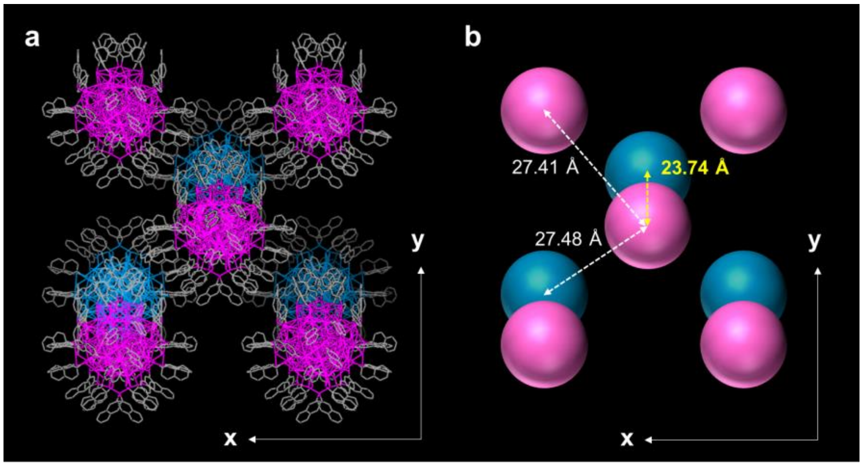

Figure S4. Interparticle distance in a Au_{103} crystal. a, Crystal packing from c-axis view. **b**, Model structure of **a** with interparticle distance. Color codes: gray = carbon; magenta and blue are used for Au and S in different enantiomers.

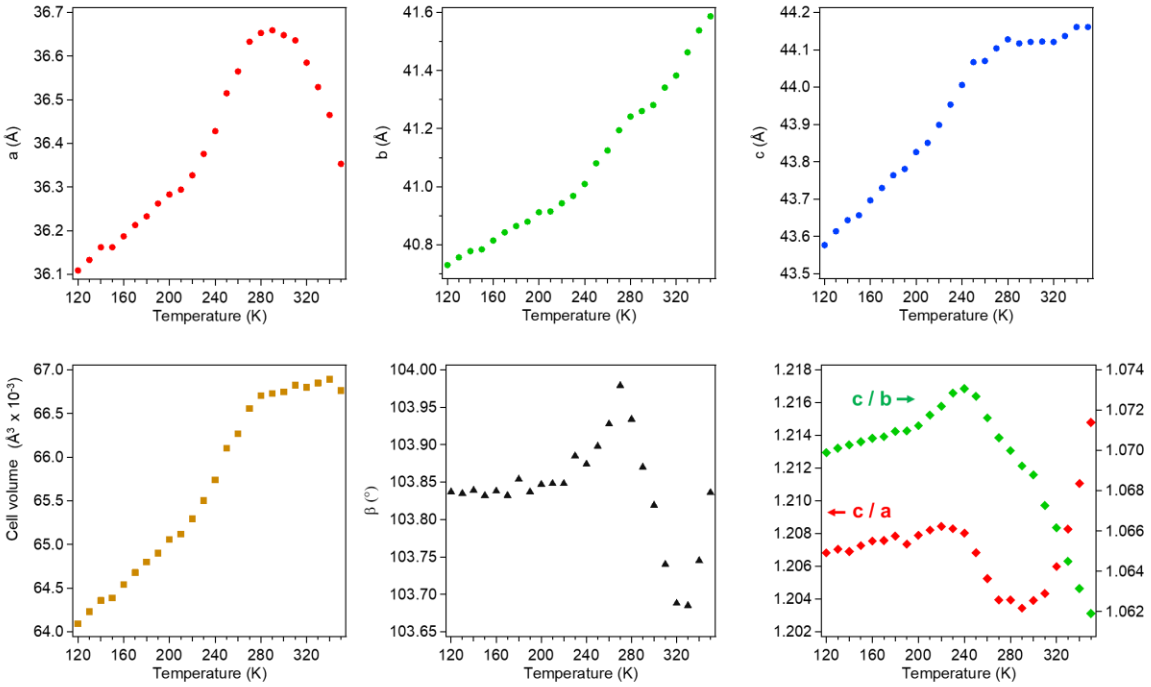

Figure S5. Variable temperature unit cell analyses by X-ray crystallography for a Au_{103} crystal.

Supporting Tables:

Table S1. Crystal data and structure refinement for Au_{103} at 300K.

Identification code	Au103-300K
Empirical formula	$C_{410}H_{287}Au_{103}S_{43}$
Formula weight	26879.51
Temperature/K	300.01(10)
Crystal system	monoclinic
Space group	C2/c
a/Å	36.7530(8)
b/Å	41.254(14)
c/Å	44.0875(8)
α/°	90
β/°	103.982(2)
γ/°	90
Volume/$Å^3$	64865(22)
Z	4
$\rho_{calc}g/cm^3$	2.752
μ/mm^{-1}	44.039
F(000)	46288.0
Crystal size/mm^3	0.276 × 0.072 × 0.054
Radiation	CuKα (λ = 1.54184)
2Θ range for data collection/°	6.552 to 88.986
Index ranges	-33 ≤ h ≤ 32, -37 ≤ k ≤ 37, -31 ≤ l ≤ 40
Reflections collected	116814
Independent reflections	25513 [R_{int} = 0.1183, R_{sigma} = 0.1104]
Data/restraints/parameters	25513/2456/1022
Goodness-of-fit on F^2	1.079
Final R indexes [I>=2σ (I)]	R_1 = 0.0846, wR_2 = 0.2251
Final R indexes [all data]	R_1 = 0.1346, wR_2 = 0.2690
Largest diff. peak/hole / e $Å^{-3}$	3.28/-2.52

Table S2. Crystal data and structure refinement for Au_{103} at 210K.

Identification code	Au103-210K
Empirical formula	$C_{410}H_{287}Au_{103}S_{43}$
Formula weight	26879.51
Temperature/K	209.99(10)
Crystal system	monoclinic
Space group	C2/c
a/Å	36.2023(7)
b/Å	41.1857(8)
c/Å	44.1204(9)
α/°	90
β/°	104.399(2)
γ/°	90
Volume/Å^3	63718(2)
Z	4
ρ_{calc}g/cm^3	2.802
μ/mm^{-1}	44.832
F(000)	46288.0
Crystal size/mm^3	0.24 × 0.04 × 0.04
Radiation	CuKα (λ = 1.54178)
2Θ range for data collection/°	6.914 to 151.214
Index ranges	$-42 \leq h \leq 44$, $-42 \leq k \leq 49$, $-54 \leq l \leq 48$
Reflections collected	178849
Independent reflections	57931 [R_{int} = 0.1111, R_{sigma} = 0.1120]
Data/restraints/parameters	57931/2516/1044
Goodness-of-fit on F^2	0.963
Final R indexes [I>=2σ (I)]	R_1 = 0.0858, wR_2 = 0.2192
Final R indexes [all data]	R_1 = 0.1331, wR_2 = 0.2573
Largest diff. peak/hole / e Å^{-3}	3.67/-2.99

Supporting References:

S1. Clark, R. C. & Reid, J. S. *Acta Cryst.* **A51** (1995), 887-897.

S2. Version 1.171.38.46 (2015). Rigaku Oxford Diffraction.

S3. Sheldrick, G. M. *Acta Cryst.* **A71** (2015), 3-8.

S4. Higaki, T. *et al. J. Am. Chem. Soc.* **139** (2017), 9994–10001.

S5. Sheldrick, G. M. *Acta Cryst.* **C71** (2015), 3-8.

S6. Dolomanov, O. V., Bourhis, L. J., Gildea, R. J., Howard, J. A. K. & Puschmann, H. *J. Appl. Cryst*. **42** (2009), 339-341.

S7. Spek, A. *Acta Cryst*. **D65** (2009), 148-155.

S8. Van der Sluis, P. & Spek, A. L. *Acta Cryst*. **A46** (1990), 194-201.

S9. CrystalMaker Software Ltd, Oxford, England *(www.crystalmaker.com)*.

S10. Egold, H., Schwarze, D. & Flörke, U. *J. Chem. Soc. Dalt. Trans.* (1999), 3203–3207.
